# Inferring Protective CD8^+^ T-Cell Epitopes for NS5 Protein of Four Serotypes of Dengue Virus Chinese Isolates Based on HLA-A, -B and -C Allelic Distribution: Implications for Epitope-Based Universal Vaccine Design

**DOI:** 10.1371/journal.pone.0138729

**Published:** 2015-09-18

**Authors:** Jiandong Shi, Jing Sun, Meini Wu, Ningzhu Hu, Jianfan Li, Yanhan Li, Haixuan Wang, Yunzhang Hu

**Affiliations:** 1 Institute of Medical Biology, Chinese Academy of Medical Sciences and Peking Union Medical College, Kunming, 650118, China; 2 Yunnan Provincial Key Laboratory of Arbo Infectious Disease Control Research (Preparing), Institute of Medical Biology, Chinese Academy of Medical Sciences, Kunming, 650118, China; Singapore Immunology Network, Agency for Science, Technology and Research (A*STAR), SINGAPORE

## Abstract

Dengue is one of the most globally serious vector-borne infectious diseases in tropical and subtropical areas for which there are currently no effective vaccines. The most highly conserved flavivirus protein, NS5, is an indispensable target of CD8^+^ T-cells, making it an ideal vaccine design target. Using the Immune Epitope Database (IEDB), CD8^+^ T-cell epitopes of the dengue virus (DENV) NS5 protein were predicted by genotypic frequency of the HLA-A,-B, and-C alleles in Chinese population. Antigenicity scores of all predicted epitopes were analyzed using VaxiJen v2.0. The IEDB analysis revealed that 116 antigenic epitopes for HLA-A (21),-B (53), and-C (42) had high affinity for HLA molecules. Of them, 14 had 90.97–99.35% conversancy among the four serotypes. Moreover, five candidate epitopes, including ^200^NS5^210^ (94.84%, A*11:01), ^515^NS5^525^ (98.71%, A*24:02), ^225^NS5^232^ (99.35%, A*33:03), ^516^NS5^523^ (98.71%, A*33:03), and ^284^NS5^291^ (98.06%, A*33:03), were presented by HLA-A. Four candidate epitopes, including ^234^NS5^241^ (96.77%, B*13:01), ^92^NS5^99^ (98.06%, B*15:01, B*15:02, and B*46:01), ^262^NS5^269^ (92.90%, B*38:02), and ^538^NS5^547^ (90.97%, B*51:01), were presented by HLA-B. Another 9 candidate epitopes, including ^514^NS5^522^ (98.71%, C*01:02), ^514^NS5^524^ (98.71%, C*01:02 and C*14:02), ^92^NS5^99^ (98.06%, C*03:02 and C*15:02), ^362^NS5^369^ (44.84%, C*03:04 and C*08:01), ^225^NS5^232^ (99.35%, C*04:01), ^234^NS5^241^(96.77%, C*04:01), ^361^NS5^369^ (94.84%, C*04:01), ^515^NS5^522^ (98.71%, C*14:02), ^515^NS5^524^ (98.71%, C*14:02), were presented by HLA-C. Further data showed that the four-epitope combination of ^92^NS5^99^ (B*15:01, B*15:02, B*46:01, C*03:02 and C*15:02), ^200^NS5^210^ (A*11:01), ^362^NS5^369^ (C*03:04, C*08:01), and ^514^NS5^524^ (C*01:02, C*14:02) could vaccinate >90% of individuals in China. Further *in vivo* study of our inferred novel epitopes will be needed for a T-cell epitope-based universal vaccine development that may prevent all four China-endemic DENV serotypes.

## Introduction

Dengue virus (DENV) can cause dengue hemorrhagic fever (DHF) and dengue shock syndrome (DSS), globally important mosquito-borne diseases [1, 2]. These are among the most serious epidemic arbovirus diseases and endemic in tropical and subtropical regions of the word. The causative viruses are members of the genus *Flavivirus* within the family Flaviviridae and can be grouped into four antigenically distinct serotypes (DENV1-4) that share 67–75% sequence homology [3, 4]. DENV is transmitted to humans through the bites of infected *Aedes aegypti* and *Aedes albopictus* mosquitoes. Nearly half of the world’s population is under risk of contracting dengue. It is estimated that up to 390 million infections occur annually worldwide with approximately 96 million symptomatic cases [5]. Despite more than 60 years of effort, no licensed vaccine is currently available. Thus, the search for a safe and effective vaccine is growing more imperative.

Dengue is hyperendemic and has become a serious public health concern in China. The first outbreak of dengue was reported in Guangdong Province of China in 1978 [6, 7]. Since then, annual DENV epidemics have occurred, followed by a dengue epidemic in Guangxi, Fujian, Zhejiang, and other areas of China. In 2014, the most serious dengue epidemic in history occurred in Guangdong province of China with a total of 48,162 infected individuals [8]. This outbreak is considered an imported epidemic from neighboring Southeast Asian countries [9, 10]. In recent years, the scope of the epidemic is further expanding from the coastal city of China to inland cities. In 2013, an outbreak of DENV occurred in Yunnan province of China with more than 2,000 infected individuals [11]. A safe and effective dengue vaccine is urgent need in China.

CD8^+^ T-cell-mediated immunity plays an important role for eliminating intracellular pathogens. Thus, eliciting robust CD8^+^ T-cell immunity is the basis for many vaccines under development. Although DENV-specific CD8^+^ T-cell responses have been extensively studied, the vast majority of studies focused on immunopathogenic role of T-cells during DENV infection [12–14]. The viewpoint from these studies is that serotype cross-reactive CD8^+^ T-cells may contribute to the immunopathogenesis of DHF/DSS. Thus, the vast majority of dengue vaccine candidates are designed to produce protective neutralizing antibodies with less regard for cellular immune responses. However, direct evidence linking T-cells to increased viremia or DENV-related pathology has not been demonstrated. Notably, recent extensive studies have demonstrated a protective role of CD4^+^ and CD8^+^ T-cells against homologous or heterotypic DENV infection in murine models [15–20]. Specifically, these studies demonstrated that CD8^+^ T-cells can control viral replication [16], prevent antibody-dependent enhancement (ADE) of infection [19], and DENV-induced CNS disease [18].

These findings are consistent with the murine model data of a recent study supporting the concept of a protective role of T-cells against DENV infection in humans. The results of this study showed that the secondary DENV infection in humans was not significantly associated with disease severity [21]. Further, another recent study provided the first comprehensive map of the CD8^+^ T-cell response to DENV in humans and support a HLA-linked protective but not pathogenic role for CD8^+^ T-cells against DENV infection in humans [22]. Collectively, these findings strongly imply a protective role for CD8^+^ T-cells against severe DENV disease in humans. Based on these studies, it is inferred that the lack of induction of a robust DENV-specific T-cell response may be a reason for the results of a recent efficacy trial of the most advanced dengue vaccine candidate, a tetravalent live-attenuated chimeric vaccine (CYD) based on the 17D-attenuated yellow fever virus backbone that showed only partial protection despite the induction of DENV-specific neutralizing antibody to each serotype in most subjects [23]. This means that the roles of T-cells in the context of DENV vaccination should not be ignored, and it raises the possibility that T-cell responses against all DENV serotypes might be beneficial or even required for vaccine protective efficacy. The advent of a T-cell epitope–based vaccine may offer an alternative that avoids ADE. Considering the important role of serotype-specific CD8^+^ T-cells in controlling DENV infection, a novel strategy for developing prophylactic and therapeutic CD8^+^ T-cell epitope-based vaccines is needed. Therefore, a T-cell epitope-based universal vaccine that induces a broad dengue-specific, multifunctional, and cross-reactive CD8^+^ T-cell responses among all four DENV serotypes may be a more promising strategy against DENV infections.

The DENV genome consists of a single-stranded RNA of 10.7 kb in length. The open reading frame codes three structural proteins [capsid (C) protein, preM protein, and envelope (E) protein] and seven nonstructural proteins (NS1, NS2a, NS2b, NS3, NS4a, NS4b and NS5) [24,25]. It has been shown that CD8^+^ T cells preferentially target the NS3 and NS5 proteins, while CD4^+^ T cells preferentially target the E, C, and NS1 proteins [26]. Notably, NS5 is the largest and the most highly conserved protein encoded by the DENV genome, with approximately 67–82% amino acid sequence identity among the four DENV serotypes [27]. Thus, NS5 proteins could be used as a promising target in the design of a T-cell epitope-based vaccine to induce DENV-specific protective T-cell responses.

A necessary condition for a peptide to be a CD8^+^ T-cell epitope is that it binds to human leukocyte antigen (HLA) molecules. However, HLA molecules are extremely polymorphic with several thousand variants and can bind distinct sets of peptides [28]. Each HLA variant is expressed at vastly variable frequencies in different ethnic groups and geographic regions [29]. This means that it appears that an extremely large and impractical number of peptides would have to be selected to enable the development of a broadly protective multi-epitope vaccine. A large number of studies focused on predicting epitopes from the E, prM, NS1, NS3, or NS5 proteins allowed the identification of T-cell epitopes in DENV [30–36]. However, the CD8^+^ T-cell epitopes of the NS5 protein of DENV Chinese isolates linked with the class I HLA allele in Chinese population have been poorly revealed. Therefore, the identification of CD8^+^ T-cell epitopes that can induce protective DENV-specific T-cell responses by a feasible immunoinformatics approach is critical and urgent for the development of a T-cell epitope-based vaccine.

In this study, based on the distribution characteristics of HLA class I alleles in Chinese population, we identified putative CD8^+^ T-cell epitopes of NS5 protein of Chinese DENV isolates using various immunoinformatics approaches. Our results provide putative protective CD8^+^ T-cell epitope candidates or their combination for the development of a T-cell epitope-based universal vaccine to effectively prevent all four DENV serotypes that are endemic in China.

## Materials and Methods

### Retrieving the protein sequences

The sequences of the NS5 protein from 155 Chinese isolates belong to all four serotypes of dengue virus (DENV-1, DENV-2, DENV-3 and DENV-4) were retrieved from the National Center for Biotechnology Information (NCBI) protein database (http://www.ncbi.nlm.nih.gov/protein/). The sequence of DENV-2 NS5 protein (accession number: KC131142.1) was used as an input for various bioinformatics tools for epitope prediction, antigenicity analysis and conservation analysis.

### HLA genotypic frequency retrieval

To improve population coverage of the CD8^+^ T-cell epitopes, it is important to screen epitopes restricted by highly prevalent HLA alleles. Genotypic frequencies of the HLA class I alleles that include HLA-A,-B and-C loci in Chinese population were retrieval from the major histocompatibility complex database (dbMHC) (http://www.ncbi.nlm.nih.gov/projects/gv/mhc/main.fcgi?cmd=init). For a broad coverage, HLA class I alleles with genotypic frequency >3% in Chinese population were selected for CD8^+^ T-cell epitope prediction. This parameter setting covers the highly prevalent HLA-A,-B, and-C alleles found in Chinese population.

### Epitope prediction

CD8^+^ T-cell epitope is the minimal amino acid sequence required for CD8^+^ T-cells activation and recognition by immune system receptors. Since the affinity of an epitope binds to the HLA molecule plays a vital role in determining its immunogenicity. Hence, high affinity between epitopes and HLA molecules tends to be associated with higher immune responsiveness. The prediction of CD8^+^ T-cell epitopes that interact with different HLA class I alleles were performed using the IEDB analysis resource (http://tools.immuneepitope.org/mhci/) Consensus tool [37], which combines predictions from ANN, aka NetMHC 3.4 [38][39], SMM [40] and Comblib [41], if any corresponding predictor is available for the molecule. Otherwise, NetMHCpan is used. This choice was motivated by the expected predictive performance of the methods in decreasing order: Consensus > ANN > SMM > NetMHCpan > CombLib. For the IEDB-recommended method, a low percentile indicated strong binding affinity to HLA molecules. The threshold of percentile rank was set at 1 in this study [42]. Based on the representative length of peptides that bind to HLA molecules, peptide lengths of 8–11 amino acids were selected for the prediction of epitope-based peptides in this study.

### Antigenicity analysis

Antigenicity is a key characteristic of the epitope that is recognized by immune system cells and/or antibodies. Thus, the antigenicity of the predicted epitopes is one of the most important criteria for epitope-based vaccine assessment. Since some of the predicted epitopes may lose antigenicity when analyzed, to ensure that the predicted epitopes could serve as a good CD8^+^ T-cell epitope, all of the epitopes were screened to assess their antigenicity. VaxiJen v2.0 (http://www.ddg-pharmfac.net/vaxijen/VaxiJen), an online web server used to predict the effective antigens and subunit vaccines, was used to identify and reevaluate T-cell epitope antigenicity. The predicted epitopes were uploaded in a plain sequence format and the virus was chosen as the target organism. The threshold level of an antigen was set at 0.5. The Vaxijen server performed well with 87% accuracy at a threshold of 0.5 antigenic score for viruses [43]. VaxiJen v2.0 allows antigen classification based on the physicochemical properties of proteins without recourse to sequence alignment. Finally, the epitopes with an antigenic score > 0.5 were selected as antigenic for the conservancy analysis.

### Conservancy and population coverage analysis

To obtain the universal T-cell epitopes of four DENV serotype variants, the conservancy of candidate epitopes should be considered prior to other criteria, even population coverage rate. As a universal T-cell epitope, it should be highly conserved in all viral variants. Hence, to determine the conservation level of the predicted epitopes among the NS5 protein sequences of the different DENV strains, the predicted epitopes were analyzed for their conservancy using the IEDB epitope conservancy tool (http://tools.immuneepitope.org/tools/conservancy/iedb_input) with a sequence identity threshold of 100%. The conservancy level of each potential epitope was calculated by seeking identities in all NS5 protein sequences of the four DENV serotype variants retrieved from the NCBI protein database. The epitopes that were 100% conserved in >90% of the sequences analyzed in four serotypes were selected as candidate epitopes. These highly conserved epitopes were selected and used to determine the population coverage by the IEDB population coverage calculation tool (http://tools.immuneepitope.org/tools/population/iedb_input). Finally, all of the selected epitopes were analyzed for similarity with human proteome using the BLAST program (http://www.ncbi.nlm.nih.gov/BLAST/) to verify that they would not trigger autoimmunity.

## Results and Discussion

### Retrial of NS5 protein sequences of four DENV serotypes

The NS5 proteins sequence of all four DENV serotypes circulating in China were retrieved in FASTA format from the NCBI protein database. A total of 155 sequences of the NS5 protein of Chinese isolates of the four DENV serotypes was obtained (S1 File, S2 File, S3 File and S4 File) and used for the further epitope analysis.

### HLA class I alleles analysis

Since specific HLA alleles are expressed at variable frequencies in different ethnic groups and different geographic regions. Therefore, HLA allele frequencies prevalent in dengue hyperendemic areas must be considered in vaccine design. Here we focused on DENV-specific T-cell epitopes that are associated with the highly prevalent HLA alleles in a Chinese population. To this end, the genotypic frequency of the highly prevalent HLA class I alleles found in this Chinese population (>3%) that include HLA-A,-B, and-C loci were obtained from the dbMHC database. As a result, seven HLA-A alleles (A*02:01, A*02:03, A*02:06, A*02:07, A*11:01, A*24:02, and A*33:03), eight HLA-B alleles (B*13:01, B*15:01, B*15:02, B*38:02, B*40:01, B*46:01, B*51:01, and B*58:01), and eight HLA-C alleles (C*01:02, C*03:02, C*03:03, C*03:04, C*04:01, C*08:01, C*14:02, and C*15:02) were obtained (Table 1).

**Table 1:** Frequency of HLA class I alleles (>3%) in Chinese population.

Allele	Frequency
HLA-A*02:01	0.053
HLA-A*02:03	0.108
HLA-A*02:06	0.035
HLA-A*02:07	0.094
HLA-A*11:01	0.277
HLA-A*24:02	0.172
HLA-A*33:03	0.115
HLA-B*13:01	0.082
HLA-B*15:01	0.044
HLA-B*15:02	0.071
HLA-B*38:02	0.071
HLA-B*40:01	0.149
HLA-B*46:01	0.115
HLA-B*51:01	0.050
HLA-B*58:01	0.089
HLA-C*01:02	0.169
HLA-C*03:02	0.087
HLA-C*03:03	0.041
HLA-C*03:04	0.128
HLA-C*04:01	0.044
HLA-C*08:01	0.126
HLA-C*14:02	0.036
HLA-C*15:02	0.037

### Prediction and antigenic analysis of CD8^+^ T-cell epitopes

CD8^+^ T-cell responses play a substantial role in eliminating DENV infected cells that cannot be managed by antibody responses. An effective vaccine that can provide protection against dengue virus infection requires robust, broad, and multi-functional CD8^+^ T-cell responses. Therefore, the CD8^+^ T-cell epitopes that bind to different HLA class I alleles with varying affinities must first be identified. Here a large number of the antigenic epitopes with a high binding affinity score of <1 percentile and an antigenicity score > 0.5 were obtained from NS5 proteins of DENV Chinese isolates against HLA-A,-B, and-C alleles. Most epitopes bind with high affinity to single HLA-A,-B, or-C molecules. As a consequence, a total of 21 antigenic epitopes were obtained against the seven alleles of the HLA-A loci (Table 2). A total of 53 antigenic epitopes were obtained against the eight alleles of the HLA-B loci (Table 3), while 42 antigenic epitopes were obtained against the eight alleles of the HLA-C loci (Table 4). Surprisingly, none of the epitopes bind to HLA-A*02:07. Notably, some epitopes, like ^92^AMTDTTPF^99^ (B*15:01, B*15:02, B*46:01, C*03:02, and C*15:02), ^515^MYFHRRDLRL^524^ (A*24:02 and C*14:02), ^225^WYMWLGAR^232^ (A*33:03 and C*04:01), ^234^LEFEALGF^241^ (B*13:01 and C*04:01), ^514^LMYFHRRDLRL^524^ (C*01:02 and C*14:02), and ^362^FTNMEAQL^369^ (C*03:04 and C*08:01) can be presented by multiple HLA molecules, suggesting that they can cover a broader population and may be better epitope vaccine candidates. Further, the potential of the epitope and HLA binding is essential in the assessment of the immunogenic potential of epitopes. Hence, as a ligand of the HLA-A molecule, epitopes ^225^WYMWLGAR^233^ and ^224^IWYMWLGARF^233^ presented by HLA-A*24:02 were the best binders based on their 0.2 percentile. As ligands of HLA-B molecules, epitopes ^89^TQMAMTDTTPF^99^ and ^530^CSAVPSHW^537^, which were presented by HLA-B*15:01 and HLA-B*58:01, respectively, were the best binders based on their 0.1 percentile. Likewise, as a ligand of HLA-C molecule, epitope ^91^MAMTDTTPF^99^ presented by HLA-C*03:02 was the best binder based on its 0.1 percentile. Additionally, HLA-A*24:02 has the highest number of binding epitopes (9/21), followed by A*33:03 (6/21) in the HLA-A allele. HLA-B*13:01 and HLA-B*58:01 presented the same most frequent epitopes (13/53), followed by B*15:01 (12/53), B*46:01 (12/53), B*38:02 (11/53), and B*15:02 (9/53) in the HLA-B alleles. Moreover, HLA-C*04:01 presented the most frequent epitopes (17/42), followed by C*14:02 (13/42), C*01:02 (8/42), C*03:02 (8/42), and C*15:02 (7/42) in the HLA-C alleles. These calculations were made on the basis of HLA genotypic frequencies assuming non-linkage disequilibrium between HLA loci. Overall, these results provide *in silico* insight for class I HLA allele-restricted CD8^+^ T-cell epitopes against the NS5 protein of DENV in Chinese population.

**Table 2:** HLA-A restricted epitopes of the NS5 protein and their binding affinity, antigenicity and conservation (in percentages) in different serotypes.

No.	Allele	Start^a^	End^a^	Peptide length	Sequence	Method used	Percentile rank	Antigenecity score	Percent of protein sequence matches at identity ≥ 100%
001	HLA-A*02:01	553	562	10	WMTTEDMLTV	Consensus (ann/smm)	1.0	0.6668	73.55% (114/155)
002	HLA-A*02:03	599	607	9	SLIGLTSRA	Consensus (ann/smm)	0.85	2.0423	68.39% (106/155)
003	HLA-A*02:03	553	562	10	WMTTEDMLTV	Consensus (ann/smm)	0.65	0.5568	73.55% (114/155)
004	HLA-A*02:06	553	562	10	WMTTEDMLTV	Consensus (ann/smm)	0.7	0.5568	73.55% (114/155)
005	HLA-A*11:01	338	346	9	TVMDIISRR	Consensus (ann/smm)	0.55	1.0053	10.97% (17/155)
006	HLA-A*11:01	125	133	9	KITAEWLWK	Consensus (ann/smm)	0.95	0.9815	10.97% (17/155)
007	HLA-A*11:01	317	326	10	AIFRLTYQNK	Consensus (ann/smm)	0.5	1.0707	1.94% (3/155)
***008***	HLA-A*11:*01*	***200***	***210***	***11***	***CVYNMMGKREK***	***Consensus (ann/smm)***	***0*.*65***	***0*.*6848***	***94*.*84% (147/155)***
009	HLA-A*24:02	225	233	9	WYMWLGARF	Consensus (ann/smm)	0.2	0.9423	32.26% (50/155)
010	HLA-A*24:02	224	233	10	IWYMWLGARF	Consensus (ann/smm)	0.2	0.7264	32.26% (50/155)
011	HLA-A*24:02	227	236	10	MWLGARFLEF	Consensus (ann/smm)	0.25	1.0434	32.26% (50/155)
012	HLA-A*24:02	225	234	10	WYMWLGARFL	Consensus (ann/smm)	0.3	0.6068	32.26% (50/155)
013	HLA-A*24:02	544	553	10	TWSIHATHEW	Consensus (ann/smm)	0.5	1.1355	9.03% (14/155)
***014***	HLA-A*24:*02*	***515***	***524***	***10***	***MYFHRRDLRL***	***Consensus (ann/smm)***	***0*.*55***	***1*.*6075***	***98*.*71% (153/155)***
015	HLA-A*24:02	449	458	10	GWNDWTQVPF	Consensus (ann/smm)	0.65	0.9735	9.68% (15/155)
016	HLA-A*24:02	232	241	10	RFLEFEALGF	Consensus (ann/smm)	0.95	1.7726	32.26% (50/155)
017	HLA-A*24:02	226	236	11	YMWLGARFLEF	Consensus (ann/smm)	0.95	1.0316	32.26% (50/155)
018	HLA-A*33:03	513	520	8	SLMYFHRR	netmhcpan	0.2	1.5676	80.00% (124/155)
***019***	HLA-A*33:*03*	***225***	***232***	***8***	***WYMWLGAR***	***netmhcpan***	***0*.*4***	***1*.*2415***	***99*.*35% (154/155)***
020	HLA-A*33:03	512	519	8	WSLMYFHR	netmhcpan	0.4	0.7229	80.00% (124/155)
***021***	HLA-A*33:*03*	***516***	***523***	***8***	***YFHRRDLR***	***netmhcpan***	***0*.*4***	***1*.*6748***	***98*.*71% (153/155)***
022	HLA-A*33:03	395	402	8	NWLVRVGR	netmhcpan	0.6	1.2717	3.87% (6/155)
***023***	HLA-A*33:*03*	***284***	***291***	***8***	***DTAGWDTR***	***netmhcpan***	***0*.*7***	***1*.*7765***	***98*.*06% (152/155)***

^a^The epitopes location in NS5 protein are from accession number: KC131142.1.

Bold and italic- indicates the percentage of epitope that is 100% conserved in more than 90% of the sequences analysed in four serotypes.

**Table 3:** HLA-B restricted epitopes of the NS5 protein and their binding affinity, antigenicity and conservation (in percentages) in different serotypes.

No.	Allele	Start^a^	End^a^	Peptide length	Sequence	Method used	Percentile rank	Antigenecity score	Percent of protein sequence matches at identity ≥ 100%
***001***	HLA-B*13:01	***234***	***241***	***8***	***LEFEALGF***	***netmhcpan***	***0.2***	***2.1459***	***96.77% (150/155)***
002	HLA-B*13:01	128	135	8	AEWLWKEL	netmhcpan	0.3	0.8381	10.97% (17/155)
003	HLA-B*13:01	510	517	8	QMWSLMYF	netmhcpan	0.4	0.5350	80.65% (125/155)
004	HLA-B*13:01	226	233	8	YMWLGARF	netmhcpan	0.6	0.8453	32.26% (50/155)
005	HLA-B*13:01	371	378	8	RQMEGEGV	netmhcpan	0.8	0.5155	77.42% (120/155)
006	HLA-B*13:01	19	26	8	SETPNLDI	netmhcpan	0.9	0.5435	5.16% (8/155)
007	HLA-B*13:01	234	242	9	LEFEALGFL	netmhcpan	0.5	1.7009	78.71% (122/155)
008	HLA-B*13:01	19	27	9	SETPNLDII	netmhcpan	0.7	0.5209	5.16% (8/155)
009	HLA-B*13:01	520	529	10	RDLRLAANAI	netmhcpan	0.7	0.8520	29.03% (45/155)
010	HLA-B*13:01	382	391	10	IQHLTVTEEI	netmhcpan	0.9	0.7084	9.68% (15/155)
011	HLA-B*13:01	580	589	10	VESWEEIPYL	netmhcpan	1	0.5190	10.97% (17/155)
012	HLA-B*13:01	89	99	11	TQMAMTDTTPF	netmhcpan	0.2	0.9856	77.42% (120/155)
013	HLA-B*13:01	226	236	11	YMWLGARFLEF	netmhcpan	0.3	1.0316	32.26% (50/155)
***014***	HLA-B*15:01	***92***	***99***	***8***	***AMTDTTPF***	***ann***	***0.2***	***0.6985***	***98.06% (152/155)***
015	HLA-B*15:01	226	233	8	YMWLGARF	ann	0.5	0.8453	32.26% (50/155)
016	HLA-B*15:01	142	149	8	RMCTREEF	ann	0.6	1.0232	10.97% (17/155)
017	HLA-B*15:01	510	517	8	QMWSLMYF	ann	0.6	0.5350	80.65% (125/155)
018	HLA-B*15:01	91	99	9	MAMTDTTPF	Consensus (ann/comblib_sidney2008/smm)	0.2	0.6735	77.42% (120/155)
019	HLA-B*15:01	228	236	9	WLGARFLEF	Consensus (ann/comblib_sidney2008/smm)	0.6	1.5566	32.26% (50/155)
020	HLA-B*15:01	90	99	10	QMAMTDTTPF	Consensus (ann/smm)	0.3	0.9860	77.42% (120/155)
021	HLA-B*15:01	89	99	11	TQMAMTDTTPF	ann	0.1	0.9856	77.42% (120/155)
022	HLA-B*15:01	226	236	11	YMWLGARFLEF	ann	0.3	1.0316	32.26% (50/155)
023	HLA-B*15:01	330	340	11	VQRPTPRGTVM	ann	0.3	0.8415	9.68% (15/155)
024	HLA-B*15:02	226	233	8	YMWLGARF	ann	0.2	0.8453	32.26% (50/155)
025	HLA-B*15:02	622	629	8	SLIGNEEY	ann	0.3	0.8841	10.32% (16/155)
***026***	HLA-B*15:02	***92***	***99***	***8***	***AMTDTTPF***	***ann***	***0.7***	***0.6985***	***98.06% (152/155)***
027	HLA-B*15:02	510	517	8	QMWSLMYF	ann	1	0.5350	80.65% (125/155)
028	HLA-B*15:02	90	99	10	QMAMTDTTPF	ann	0.3	0.9860	77.42% (120/155)
029	HLA-B*15:02	89	99	11	TQMAMTDTTPF	ann	0.3	0.9856	77.42% (120/155)
030	HLA-B*15:02	226	239	11	YMWLGARFLEF	ann	0.3	1.0316	32.26% (50/155)
031	HLA-B*15:02	622	632	11	SLIGNEEYTDY	ann	0.3	0.9393	10.32% (16/155)
032	HLA-B*15:02	330	340	11	VQRPTPRGTVM	ann	0.9	0.8415	9.68% (15/155)
033	HLA-B*38:02	124	131	8	MKITAEWL	netmhcpan	1	0.5377	10.97% (17/155)
***034***	HLA-B*38:02	***262***	***269***	***8***	***LHKLGYIL***	***netmhcpan***	***1***	***0.7094***	***92.90% (144/155)***
035	HLA-B*38:02	383	391	9	QHLTVTEEI	netmhcpan	0.2	0.9107	9.68% (15/155)
036	HLA-B*38:02	42	50	9	WHYDQDHPY	netmhcpan	0.4	0.5520	9.03% (14/155)
037	HLA-B*38:02	91	99	9	MAMTDTTPF	netmhcpan	0.8	0.6735	77.42% (120/155)
038	HLA-B*38:02	89	99	11	TQMAMTDTTPF	netmhcpan	0.3	0.9856	77.42% (120/155)
039	HLA-B*38:02	383	393	11	QHLTVTEEIAV	netmhcpan	0.4	0.8018	9.68% (15/155)
040	HLA-B*38:02	535	545	11	SHWVPTSRTTW	netmhcpan	0.5	1.4792	10.97% (17/155)
041	HLA-B*38:02	626	636	22	NEEYTDYMPSM	netmhcpan	0.8	0.6913	10.32% (16/155)
042	HLA-B*38:02	480	490	11	NQDELIGRARI	netmhcpan	0.9	0.6578	14.19% (22/155)
043	HLA-B*38:02	226	236	11	YMWLGARFLEF	netmhcpan	1	1.0316	32.26% (50/155)
044	HLA-B*40:01	250	257	8	RENSLSGV	Consensus (ann/smm)	0.75	0.8542	28.39% (44/155)
045	HLA-B*40:01	234	242	9	LEFEALGFL	Consensus (ann/smm)	0.2	1.7009	78.71% (122/155)
046	HLA-B*40:01	19	27	9	SETPNLDII	Consensus (ann/smm)	0.85	0.5209	5.16% (8/155)
047	HLA-B*40:01	580	589	10	VESWEEIPYL	Consensus (ann/smm)	0.5	0.5109	10.97% (17/155)
048	HLA-B*40:01	182	191	10	WELVDKERNL	Consensus (ann/smm)	0.85	1.4932	10.97% (17/155)
***049***	HLA-B*46:01	***92***	***99***	***8***	***AMTDTTPF***	***ann***	***0.3***	***0.6985***	***98.06% (152/155)***
050	HLA-B*46:01	226	233	8	YMWLGARF	ann	0.4	0.8453	32.26% (50/155)
051	HLA-B*46:01	510	517	8	QMWSLMYF	ann	0.7	0.5350	80.65% (125/155)
052	HLA-B*46:01	467	474	8	IMKDGRVL	ann	1	0.6354	10.97% (17/155)
053	HLA-B*46:01	629	636	8	YTDYMPSM	ann	1	0.8868	10.97% (17/155)
054	HLA-B*46:01	91	99	9	MAMTDTTPF	Consensus (ann/smm)	0.2	0.6735	77.42% (120/155)
055	HLA-B*46:01	272	280	9	VSKKEGGAM	Consensus (ann/smm)	0.65	0.5314	10.97% (17/155)
056	HLA-B*46:01	248	257	10	FSRENSLSGV	ann	0.7	1.2193	28.39% (44/155)
057	HLA-B*46:01	90	99	10	QMAMTDTTPF	ann	0.8	0.9860	77.42% (120/155)
058	HLA-B*46:01	78	87	10	LTKPWDVIPM	ann	0.9	0.9964	32.26% (50/155)
059	HLA-B*46:01	226	236	11	YMWLGARFLEF	ann	0.2	1.0316	32.26% (50/155)
060	HLA-B*46:01	89	99	11	TQMAMTDTTPF	ann	0.8	0.9856	77.42% (120/155)
061	HLA-B*51:01	85	93	9	IPMVTQMAM	Consensus (ann/comblib_sidney2008/smm)	0.5	0.5370	10.97% (17/155)
062	HLA-B*51:01	80	88	9	KPWDVIPMV	Consensus (ann/comblib_sidney2008/smm)	0.8	1.2766	32.26% (50/155)
***063***	HLA-B*51:01	***538***	***547***	***10***	***VPTSRTTWSI***	***Consensus (ann/smm)***	***0.3***	***1.0517***	***90.97% (141/155)***
064	HLA-B*58:01	530	537	8	CSAVPSHW	ann	0.1	0.8475	10.97% (17/155)
065	HLA-B*58:01	125	132	8	KITAEWLW	ann	0.3	1.0187	10.97% (17/155)
066	HLA-B*58:01	123	130	8	LMKITAEW	ann	1	1.0313	10.97% (17/155)
067	HLA-B*58:01	545	553	9	KLMKITAEW	Consensus (ann/comblib_sidney2008/smm)	0.2	1.0764	10.97% (17/155)
068	HLA-B*58:01	91	99	9	MAMTDTTPF	Consensus (ann/comblib_sidney2008/smm)	0.3	0.6735	77.42% (120/155)
069	HLA-B*58:01	124	132	9	MKITAEWLW	Consensus (ann/comblib_sidney2008/smm)	0.3	0.7346	10.97% (17/155)
070	HLA-B*58:01	601	609	9	IGLTSRATW	Consensus (ann/comblib_sidney2008/smm)	0.4	1.5513	68.39% (106/155)
071	HLA-B*58:01	621	629	9	RSLIGNEEY	Consensus (ann/comblib_sidney2008/smm)	0.9	0.9167	10.32% (16/155)
072	HLA-B*58:01	544	553	10	TWSIHATHEW	Consensus (ann/smm)	0.6	1.1355	9.03% (14/155)
073	HLA-B*58:01	33	42	10	KIKQEHETSW	Consensus (ann/smm)	0.6	0.9160	10.97% (17/155)
074	HLA-B*58:01	123	132	10	LMKITAEWLW	Consensus (ann/smm)	0.65	0.9159	10.97% (17/155)
075	HLA-B*58:01	237	247	11	EALGFLNEDHW	Consensus (ann/smm)	0.75	1.0740	77.42% (120/155)
076	HLA-B*58:01	599	609	11	SLIGLTSRATW	Consensus (ann/smm)	0.95	1.7930	68.39% (106/155)

^a^The epitopes location in NS5 protein are from accession number: KC131142.1.

Bold and italic- indicates the percentage of epitope that is 100% conserved in more than 90% of the sequences analysed in four serotypes.

**Table 4:** HLA-C restricted epitopes of the NS5 protein and their binding affinity, antigenicity and conservation (in percentages) in different serotypes.

No.	Allele	Start^a^	End^a^	Peptide length	Sequence	Method used	Percentile rank	Antigenecity score	Percent of protein sequence matches at identity ≥100%
001	HLA-C*01:02	629	636	8	YTDYMPSM	netmhcpan	0.6	0.8868	10.97% (17/155)
002	HLA-C*01:02	91	99	9	MAMTDTTPF	netmhcpan	0.2	0.6735	77.42% (120/155)
003	HLA-C*01:02	85	93	9	IPMVTQMAM	netmhcpan	0.7	0.5370	10.97% (17/155)
***004***	HLA-C*01:*02*	***514***	***522***	***9***	***LMYFHRRDL***	***netmhcpan***	***0*.*8***	***1*.*5116***	***98*.*71% (153/155)***
005	HLA-C*01:02	84	93	10	VIPMVTQMAM	netmhcpan	0.6	0.7337	10.97% (17/155)
006	HLA-C*01:02	513	522	10	SLMYFHRRDL	netmhcpan	0.7	1.4737	80.00% (124/155)
007	HLA-C*01:02	226	236	11	YMWLGARFLEF	netmhcpan	0.8	1.0316	32.26% (50/155)
***008***	HLA-C*01:*02*	***514***	***524***	***11***	***LMYFHRRDLRL***	***netmhcpan***	***0*.*9***	***1*.*5932***	***98*.*71% (153/155)***
009	HLA-C*03:02	629	636	8	YTDYMPSM	netmhcpan	0.4	0.8868	10.97% (17/155)
***010***	HLA-C*03:*02*	***92***	***99***	***8***	***AMTDTTPF***	***netmhcpan***	***0*.*9***	***0*.*6985***	***98*.*06% (152/155)***
011	HLA-C*03:02	226	233	8	YMWLGARF	netmhcpan	1	0.8453	32.26% (50/155)
012	HLA-C*03:02	91	99	9	MAMTDTTPF	netmhcpan	0.1	0.6735	77.42% (120/155)
013	HLA-C*03:02	615	623	9	TAINQVRSL	netmhcpan	0.7	0.6391	10.97% (17/155)
014	HLA-C*03:02	545	554	10	WSIHATHEWM	netmhcpan	0.8	0.8628	9.03% (14/155)
015	HLA-C*03:02	226	236	11	YMWLGARFLEF	netmhcpan	0.5	1.0316	32.26% (50/155)
016	HLA-C*03:02	89	99	11	TQMAMTDTTPF	netmhcpan	1	0.9856	77.42% (120/155)
017	HLA-C*03:03	615	623	9	TAINQVRSL	Consensus (ann/smm)	0.6	0.6391	10.97% (17/155)
018	HLA-C*03:03	501	511	11	TACLGKSYAQM	ann	0.9	0.6954	29.03% (45/155)
019	HLA-C*03:03	474	484	11	LVVPCRNQDEL	ann	1	1.0079	14.19% (22/155)
020	HLA-C*03:04	629	636	8	YTDYMPSM	netmhcpan	0,5	0.8868	10.97% (17/155)
***021***	HLA-C*03:*04*	***362***	***369***	***8***	***FTNMEAQL***	***netmhcpan***	***1***	***0*.*7649***	***94*.*84% (147/155)***
022	HLA-C*03:04	91	99	9	MAMTDTTPF	netmhcpan	0.2	0.6735	77.42% (120/155)
023	HLA-C*03:04	615	623	9	TAINQVRSL	netmhcpan	0.4	0.6391	10.97% (17/155)
024	HLA-C*04:01	287	294	8	GWDTRITL	ann	0.2	1.6194	10.97% (17/155)
025	HLA-C*04:01	582	589	8	SWEEIPYL	ann	0.4	0.9783	10.97% (17/155)
***026***	HLA-C*04:*01*	***225***	***232***	***8***	***WYMWLGAR***	***ann***	***0*.*5***	***1*.*2415***	***99*.*35% (154/155)***
027	HLA-C*04:01	226	233	8	YMWLGARF	ann	0.5	0.8453	32.26% (50/155)
***028***	HLA-C*04:*01*	***234***	***241***	***8***	***LEFEALGF***	***ann***	***1***	***2*.*1459***	***96*.*77% (150/155)***
029	HLA-C*04:01	287	295	9	GWDTRITLE	Consensus (ann/smm)	0.25	1.4743	10.97% (17/155)
030	HLA-C*04:01	225	233	9	WYMWLGARF	Consensus (ann/smm)	0.75	0.9423	32.26% (50/155)
***031***	HLA-C*04:*01*	***361***	***369***	***9***	***TFTNMEAQL***	***Consensus (ann/smm)***	***1***	***0*.*8131***	***94*.*84% (147/155)***
032	HLA-C*04:01	450	458	9	WNDWTQVPF	Consensus (ann/smm)	0.6	0.9685	9.68% (15/155)
033	HLA-C*04:01	225	234	10	WYMWLGARFL	ann	0.2	0.6068	32.26% (50/155)
034	HLA-C*04:01	449	458	10	GWNDWTQVPF	ann	0.3	0.9735	9.68% (15/155)
035	HLA-C*04:01	224	233	10	IWYMWLGARF	ann	0.6	0.7264	32.26% (50/155)
036	HLA-C*04:01	223	233	11	AIWYMWLGARF	ann	0.7	0.5658	32.26% (50/155)
037	HLA-C*04:01	89	99	11	TQMAMTDTTPF	ann	0.9	0.9856	77.42% (120/155)
038	HLA-C*04:01	225	235	11	WYMWLGARFLE	ann	0.9	0.6782	32.26% (50/155)
039	HLA-C*04:01	226	236	11	YMWLGARFLEF	ann	0.9	1.0316	32.26% (50/155)
040	HLA-C*04:01	582	592	11	SWEEIPYLGKR	ann	0.9	1.2892	10.97% (17/155)
041	HLA-C*08:01	629	636	8	YTDYMPSM	netmhcpan	0.2	0.8868	10.97% (17/155)
***042***	HLA-C*08:*01*	***362***	***369***	***8***	***FTNMEAQL***	***netmhcpan***	***0*.*9***	***0*.*7649***	***94*.*84% (147/155)***
043	HLA-C*08:01	91	99	9	MAMTDTTPF	netmhcpan	0.3	0.6735	77.42% (120/155)
***044***	HLA-C*14:*02*	***515***	***522***	***8***	***MYFHRRDL***	***ann***	***0*.*2***	***1*.*5109***	***98*.*71% (153/155)***
045	HLA-C*14:02	247	254	8	WFSRENSL	ann	0.8	0.5342	28.39% (44/155)
046	HLA-C*14:02	232	239	8	RFLEFEAL	ann	0.9	1.2524	32.26% (50/155)
047	HLA-C*14:02	43	50	8	HYDQDHPY	ann	1	0.6739	9.03% (14/155)
048	HLA-C*14:02	226	233	8	YMWLGARF	ann	1	0.8453	32.26% (50/155)
049	HLA-C*14:02	628	636	9	EYTDYMPSM	Consensus (ann/smm)	0.35	0.8846	10.97% (17/155)
050	HLA-C*14:02	225	233	9	WYMWLGARF	Consensus (ann/smm)	0.65	0.9423	32.26% (50/155)
***051***	HLA-C*14:*02*	***515***	***524***	***10***	***MYFHRRDLRL***	***ann***	***0*.*2***	***1*.*6075***	***98*.*71% (153/155)***
052	HLA-C*14:02	225	234	10	WYMWLGARFL	ann	0.3	0.6068	32.26% (50/155)
053	HLA-C*14:02	84	93	10	VIPMVTQMAM	ann	1	0.7337	10.97% (17/155)
054	HLA-C*14:02	226	236	11	YMWLGARFLEF	ann		1.0316	32.26% (50/155)
***055***	HLA-C*14:*02*	***514***	***524***	***11***	***LMYFHRRDLRL***	***ann***	***0*.*9***	***1*.*5932***	***98*.*71% (153/155)***
056	HLA-C*14:02	89	99	11	TQMAMTDTTPF	ann	1	0.9856	77.42% (120/155)
057	HLA-C*15:02	226	233	8	YMWLGARF	ann	0.2	0.8453	32.26% (50/155)
058	HLA-C*15:02	622	629	8	SLIGNEEY	ann	0.3	0.8841	10.32% (16/155)
***059***	HLA-C*15:*02*	***92***	***99***	***8***	***AMTDTTPF***	***ann***	***0*.*7***	***0*.*6985***	***98*.*06% (152/155)***
060	HLA-C*15:02	510	517	8	QMWSLMYF	ann	1	0.5350	80.65% (125/155)
061	HLA-C*15:02	362	370	9	FTNMEAQLI	Consensus (ann/smm)	0.25	0.7034	29.68% (46/155)
062	HLA-C*15:02	154	162	9	RSNAALGAI	Consensus (ann/smm)	0.6	1.0339	9.03% (14/155)
063	HLA-C*15:02	360	370	11	NTFTNMEAQLI	ann	0.9	0.7529	29.68% (46/155)

^a^The epitopes location in NS5 protein are from accession number: KC131142.1.

Bold and italic- indicates the percentage of epitope that is 100% conserved in more than 90% of the sequences analysed in four serotypes.

### Conservancy and population coverage of CD8^+^ T-cell epitopes

As an effective vaccine formulation, the epitope-based universal vaccine must include highly conserved CD8^+^ T-cell epitopes among all DENV serotypes to induce cross-reactive T-cell responses based on the fact that the conserved epitope candidates are more likely to confer cross-protection between pathogen variants. Here, conservancy analysis revealed a total of 14 highly conserved epitopes with ≥90% protein sequence matching in a total of 155 NS5 protein sequences from four DENV serotypes of Chinese isolates. For the HLA-A allele, five of the 21 epitopes were conserved with ≥90% conservancy (Table 2), including ^200^CVYNMMGKREK^210^ (94.84%, A*11:01), ^515^MYFHRRDLRL^524^ (98.71%, A*24:02), ^225^WYMWLGAR^232^ (99.35%, A*33:03), ^516^YFHRRDLR^523^ (98.71%, A*33:03), and ^284^DTAGWDTR^291^(98.06%, A*33:03). For the HLA-B allele, four of the 53 epitopes were conserved with ≥90% conservancy (Table 3), including ^234^LEFEALGF^241^ (96.77%, B*13:01), ^92^AMTDTTPF^99^ (98.06%, B*15:01, B*15:02 and B*46:01), ^262^LHKLGYIL^269^ (92.90%, B*38:02), and ^538^VPTSRTTWSI^547^ (90.97%, B*51:01). For the HLA-C allele, nine of the 42 epitopes were conserved with ≥90% conservancy (Table 4), including ^514^LMYFHRRDL^522^ (98.71%, C*01:02), ^514^LMYFHRRDLRL^524^ (98.71%, C*14:02 and C*01:02), ^92^AMTDTTPF^99^ (98.06%, C*03:02 and C*15:02), ^362^FTNMEAQL^369^ (94.84%, C*03:04 and C*08:01), ^225^WYMWLGAR^232^ (99.35%, C*04:01), ^234^LEFEALGF^241^(96.77%, C*04:01), ^361^TFTNMEAQL^369^ (94.84%, C*04:01), ^515^MYFHRRDL^522^ (98.71%, C*14:02), and ^515^MYFHRRDLRL^524^ (98.71%, C*14:02).

Since the HLA allele frequencies vary among populations due to different genetic backgrounds, to design an effective vaccine, we should consider the candidate epitopes that specifically bind with the prevalent HLA molecules in the target population where the vaccine will be employed. Therefore, here we examined the population coverage of the proposed epitope vaccine candidate in a Chinese population. The results showed that the epitope ^92^AMTDTTPF^99^ (B*15:01, B*15:02, B*46:01, C*03:02, and C*15:02) has the highest percentage of population coverage (47.16%), followed by ^200^CVYNMMGKREK^210^ (A*11:01, 43.48%), ^362^FTNMEAQL^369^ (C*03:04 and C*08:01, 36.60%), ^514^LMYFHRRDLRL^524^ (C*01:02 and C*14:02, 33.53%), ^515^MYFHRRDLRL^524^ (A*24:02 and C*14:02, 28.69%), and ^514^LMYFHRRDL^522^ (C*01:02, 27.68%) in China (Table 5). It is worth noting that the combination of the epitopes ^92^AMTDTTPF^99^ (B*15:01, B*15:02, B*46:01, C*03:02 and C*15:02), ^200^CVYNMMGKREK^210^ (A*11:01), ^362^FTNMEAQL^369^ (C*03:04 and C*08:01), and ^514^LMYFHRRDLRL^524^ (C*01:02 and C*14:02) could vaccinate >90% of the Chinese population, suggesting that the four epitopes are better candidates for a multiple T-cell epitope-based vaccine. These highly conserved HLA restricted epitopes with acceptable population coverage could be putative epitope vaccine candidates in their combinations to elicit DENV-specific T-cell responses. Finally, to avoid the autoimmune response, all of the predicted class I HLA-binding antigenic epitopes were analyzed for their homology with human proteome, but no epitope was homologous with human proteome. Based on these results, we proposed that the combination of these highly conserved epitopes could be as universal CD8^+^ T-cell epitope vaccine candidates to induce DENV-specific T-cell responses against four DENV serotypes that are endemic in China.

**Table 5:** Population coverage rate (%) for the highly conserved epitopes that could be as multiple epitope-based universal vaccine candidates.

Epitope candidates	Position (aa) ^a^	HLA class I alleles	Population coverage (%)
AMTDTTPF	92–99	HLA-B*15:01, HLA-B*15:02, HLA-B*46:01, HLA-C*03:02, HLA-C*15:02	47.16
CVYNMMGKREK	200–210	HLA-A*11:01	43.48
FTNMEAQL	362–369	HLA-C*03:04, HLA-C*08:01	36.60
LMYFHRRDLRL	514–524	HLA-C*01:02, HLA-C*14:02	33.53
MYFHRRDLRL	515–524	HLA-A*24:02, HLA-C*14:02	28.69
LMYFHRRDL	514–522	HLA-C*01:02	27.68
WYMWLGAR	225–232	HLA-A*33:03, HLA-C*04:01	18.46
LEFEALGF	234–241	HLA-B*13:01, HLA-C*04:01	17.48
YFHRRDLR	516–523	HLA-A*33:03	9.78
DTAGWDTR	284–291	HLA-A*33:03	9.78
TFTNMEAQL	361–369	HLA-C*04:01	9.62
VPTSRTTWSI	538–547	HLA-B*51:01	7.39
MYFHRRDL	515–522	HLA-C*14:02	6.91
LHKLGYIL	262–269	HLA-B*38:02	5.22

^a^The epitopes location in NS5 protein are from accession number: KC131142.1.

## Conclusion

HLA-restricted epitopes for prophylactic or therapeutic vaccines against infectious diseases to induce a T-cell response that eliminates infected cells is a promising vaccine strategy. In this study, we identified 14 universal CD8^+^ T-cell epitope candidates using immunoinformatic approach, and they are highly conserved among all four DENV serotypes that are endemic in China. The combination of four epitopes, including ^92^AMTDTTPF^99^ (B*15:01, B*15:02, B*46:01, C*03:02 and C*15:02), ^200^CVYNMMGKREK^210^ (A*11:01), ^362^FTNMEAQL^369^ (C*03:04 and C*08:01), and ^514^LMYFHRRDLRL^524^ (C*01:02 and C*14:02), could vaccinate >90% of individuals in China. These epitopes are valuable T-cell epitope-based vaccine candidates for the development of a universal dengue vaccine that is capable of eliciting specific and robust protective T-cell responses against four DENV serotype variants. In conclusion, our study highlights that it is possible to design an epitope-based universal vaccine against all four DENV serotypes based on protective CD8^+^ T-cell-mediated cellular immune responses.

## Supporting Information

S1 File All available NS5 protein sequences of dengue virus serotype 1 Chinese isolates. (TXT)

S2 File All available NS5 protein sequences of dengue virus serotype 2 Chinese isolates. (TXT)

S3 File All available NS5 protein sequences of dengue virus serotype 3 Chinese isolates. (TXT)

S4 File All available NS5 protein sequences of dengue virus serotype 4 Chinese isolates. (TXT)
